# Creation of Superhydrophobic Coatings Based on MWCNTs Xerogel

**DOI:** 10.3390/nano9111584

**Published:** 2019-11-08

**Authors:** Marat Eseev, Andrey Goshev, Sergey Kapustin, Yuliana Tsykareva

**Affiliations:** Department of Fundamental and Applied Physics, Northern Arctic Federal University named after M. V. Lomonosov, Severnaya Dvina Emb. 17, 163002 Arkhangelsk, Russia; agoshev@hotmail.com (A.G.); hare22@yandex.ru (S.K.); yu.cykareva@narfu.ru (Y.T.)

**Keywords:** multi-walled carbon nanotubes, xerogel, superhydrophobic coatings, water contact angle, electrophysical properties

## Abstract

The creation of hydrophobic anti-icing and self–cleaning coatings is a relevant task for many industrial sectors. The potential field of application includes production of liquid and gas separators and filters, the field of textiles and clothing, construction and new materials, optical and microelectronic devices, the field of automobile construction and maritime shipping as well as energy and agriculture. The article suggests a new approach to the creation of superhydrophobic anti-icing coatings, by drawing peeled multi-walled carbon nanotubes (MWCNTs) to the sample surface. This method allows you to combine the necessary factors: Low surface energy, micro-nano-roughness and hierarchical multi-scale. The authors investigated the dependence of the wetting angle of such a surface on the model of MWCNT, fractional composition and the polarity of the dissolvent. The suggested approach can be used to create superhydrophobic coatings with the additional function of removing static charge and heating the surface, which can be used in the field of energetics for protection against freezing of wind turbine blades and aircraft surfaces.

## 1. Introduction

Superhydrophobic coatings are a contemporary topic; because such coatings are resistant to pollution, icing and corrosion, their use reduces maintenance and repair costs and thus prolongs product lifetimes. Therefore, successful development of self-cleaning superhydrophobic materials is a very important technological task which should reduce wear and corrosion caused by interaction of a surface with moisture, and increase the service life of the material.

Superhydrophobic coatings have a wide range of applications in such areas as: self-cleaning surfaces, microscale liquid devices, separators, textiles, automotive parts, construction, agriculture, energy (wind generator protection from freezing, self-cleaning solar cells), optical and microelectronic devices (cameras, mobile phones, lenses, optical sensors), and the maritime industry (anti-fractional and anticorrosion protection of propeller screws).

Thomas Young first examined and described the forces acting on a drop of liquid more than two hundred years ago [1]. The creation of a superhydrophobic surface became relevant about two decades ago, thanks to the work of Wenzel, Cassie and Baxter. Since then, the development of new types of superhydrophobic surfaces has become increasingly relevant, and research [2,3,4,5] continues using new materials, nanoparticles and polymers. Various methods are used for obtaining this kind of coating, including chemical etching, lithography, deposition of nanoparticles and the use of the “slippery porous surfaces soaked in a liquid” (SLIP) effect; some of them are presented in a brief overview below.

It is well established that three factors are needed to create a superhydrophobic coating: low surface energy, micron–scale roughness and hierarchical multi-scale [2,5,6,7,8]. The criterion for superhydrophobicity is a water contact angle of more than 150°.

Currently three methods are known to increase the hydrophobicity of a surface: the chemical method, the application of the lotus effect, and the SLIP effect. It is possible to use various combinations of the above-listed methods.

The chemical method of obtaining hydrophobic coatings is based on the preparation or modification of the surface by non-polar chemical functional groups. Kumar et al. [9] assume surface hydrophilicity is attributed to the presence of hydroxyl (–OH), carboxyl (–COOH) or sulphonic (–SO_3_H) groups. The increase in hydrophobicity is related to the increase of the number of nonpolar groups such as methyl (–CH_3_), ethyl (–CH_3_CH_2_), as well as fluorination. The application of such a coating is a technologically simple process, but this approach does not enable the achievement of a high water contact angle.

A study of the water-repellent properties of plant leaves was presented by Otten et al. [5]. It was shown that the hydrophobicity of some leaves could be explained by taking into account different scales of surface roughness, and sometimes additional elastic structures covering the leaf surface. Both concepts were illustrated by two different types of plant leaves, as well as a modelled surface with aggregates of carbon nanotubes on it. The natural superhydrophobicity of the lotus leaf itself is associated with the presence of an innate hydrophobic epicuticular wax and isotropic micron–large-scale roughness of leaf hairs (papillae) [10,11]. In nature, this effect is called the “lotus effect” and occurs when dew appear on certain types of plant leaves (although in the case of the lotus leaf itself, the water contact angle is 152°).

Inspired by the idea of repulsing colliding liquids in natural systems such as the “Nepenthes jar”, Wong et al. [12] produced synthetic liquid-repelling coatings soaked in a special lubricant. Such coatings are called “slippery porous surfaces soaked in a liquid” (SLIPS); they consist of a lubricating fluid film fixed by a micro–nanoporous substrate. The water contact angle is mainly determined by the adhesion of the lubricant and the liquid, which allows small sliding angles to be achieved. In order to achieve a longer time duration of the functioning of the coating it is necessary to create a microporous substrate and increase its lubricant affinity through functionalization.

Of the various approaches to the creation of superhydrophobic coatings from CNTs, the growing of vertically oriented arrays is common to find in articles. Lau et al. [3] obtained a water contact angle of 161° using a vertically oriented array of carbon nanotubes 10–15 μm in length (a nanotube forest). However, the position of the droplets was unstable and they seeped into the CNT array within a few minutes.

A vertically-aligned CNT array was coated with hydrophobic poly (tetrafluoroethylene) (PTFE) coating. This would enable significantly increasing its resistance to microdrops, decreasing the wetting of the side surfaces of CNTs. Yung et al. [13] study the light-absorbing properties of a vertically-aligned CNT array and it is noted that the proposed coating has water contact angle of 130°. Furthermore, a plasma modification of the CNT array was performed. Oxidation led to the appearance of superhydrophilic properties, and CF_4_ treatment increased the water contact angle to 154–159°. The superhydrophobic properties of the proposed coating haven’t been fully investigated in the article. For example, the authors don’t indicate the slip start angle value of the drop.

However, there are other methods of obtaining similar surfaces. One of them is the insertion of carbon nanotubes in polymer films that cover the surface. CNTs were copolymerized with thiophene/aryl compounds by Krishnamurthy et al. [14]. The films obtained were applied to leather and glass surfaces. Their properties have proven to be easily reproducible, and the water contact angle was 155° and 163°, respectively.

Zhang et al. [15] report a method for fabricating carbon nanotube (CNT)/polydimethylsiloxane (PDMS) composite superhydrophobic coatings. With toluene as a solvent, the coating is obtained directly by spray of CNT/PDMS/toluene suspension. The necessity for such a selection of component ratios that provides a high level of nano-roughness was demonstrated.

Wang et al. [16] reached a water contact angle of 152° was at a slip start angle of the drop of 2°. The superhydrophobic thin films with hierarchical structure are fabricated by spraying an environmentally friendly aqueous dispersion containing carbon nanotubes and PTFE resin on silicon wafer.

Liu et al. [17] produced an artificial lotus leaf on a cotton surface. They used pure multi-walled carbon nanotubes (MWCNT) and modified multi-walled carbon nanotubes (PBA–g–MWCNT) as building blocks to mimic the surface texture of a lotus leaf. The resulting wetting angle was greater than 150°.

Superhydrophobic coating made of polyphenylene sulfide (PPS) and a mixture of CNTs with graphene without using fluorine was suggested by Lv et al. [18]. The coating is applied by simple spraying and is resistant to salt and wear.

In [19], an interesting two-stage method was proposed for creating a superhydrophobic surface. Initially, a periodic relief was created on a silicon wafer, representing the micropillar array. Then, non-oriented CNTs were grown on it using the chemical vapor deposition (CVD) method. Varying the geometry of the surface, the authors were able to achieve a water contact angle of 153–155° degrees, with a slip start angle of the drop of 3–5° degrees. Microdroplets of liquid that could not be stopped by CNTs accumulated on their smooth lateral surface, gradually wetting them. As a result, superhydrophobic properties would be lost if a flat silicon surface was used. However, the periodic relief allows them to stabilize. The drawback of this method is the high complexity of the manufacture of the coating.

Rafiee et al. [20] suggest changing the wettability of the coating with an additive of graphene. A coating with water as a solvent showed hydrophobic properties, and a coating based on acetone—hydrophilic ones. It is also possible to control the value of the wetting angle through mixing solvents in certain ratios. An interesting approach to create an adaptive material with an adjustable degree of hydrophobicity was explored by Li et al. [21]. A composite membrane made of CNTs with polyurethane has demonstrated a change in the slip start angle and the adhesion to the droplet upon stretching the membrane. A possibility of obtaining a superhydrophobic coating based on aerogel of silica and armed with CNTs was demonstrated by Lamy-Mendes et al. [22]. In order to improve the mechanical properties, the CNTs were salinized. The superhydrophobic composite coating has demonstrated high wear resistance, thermal resistance and corrosion resistance under severe operating conditions. Papers [23,24,25,26,27,28,29] have been also dedicated to actively studying the physical properties of a coating based on CNTs, graphene, carbon black and xerogel nanofibers.

Note that despite the abundance of methods of obtaining superhydrophobic surfaces, few among those using carbon nanomaterials receive industrial implementation. This is the reason for finding a simple method of application of superhydrophobic coatings with a low cost of production costs.

## 2. Materials and Methods

Carbon nanotubes (CNTs) are non–polar hydrophobic structures with a high aspect ratio. They are strong, wear–resistant objects, have high thermal and electrical conductivity, as well as prone to the formation of porous agglomerates with a developed surface. Such characteristics allow to provide the required hydrophobic properties of the object and open up a wide field for experiments. Note that today the lowest cost, and, therefore, the most popular (in the applied sense) method for the synthesis of CNT is the CVD method. The cost of the MWCNT obtained by this method today may be less than one dollar per gram, which in turn opens the opportunities to the applied aspect of their use and the commercialization of materials with their participation. To prepare and study the properties of superhydrophobic surfaces, the authors used the MWCNTs obtained by the CVD method CNTs were manufactured by the «NanoTechCenter» company, Tambov, Russia. Characteristics are shown in Table 1.

### 2.1. Xerogel Preparation

To study the dependence of the wetting angle on the size of the CNT agglomerate, powder was prepared from milled xerogel based on CNTs. The advantage of this kind of approach is due to the good strength of its micrograins and the constancy of the morphology, which is weakly dependent on external influences.

The xerogel was prepared from Taunit–M and Taunit–MD nanotubes. Xerogel based on Taunit CNTs has not been studied due to its high brittleness. The high aspect ratio of CNTs allows the production a xerogel even without the use of a binder polymer. For this purpose, a dispersed solution of MWCNT in isopropyl/ethanol alcohol was prepared in a ratio of 1:1 by volume. Next, the nanotubes were subjected to ultrasonic dispersion with constant stirring for an hour. The resulting mass was dried for 5–6 days at room temperature under vacuum. At the final stage, the xerogel was cut into small pieces and dried at 80 degrees for about 5 h. To obtain crumbs of fractions, the xerogel was milled in a mortar and sieved through a set of sieves.

### 2.2. Obtaining the Coating

The coating was applied the following way: portions of powder (milled xerogel) were sprinkled over two steel samples covered with carbon adhesive tape, then the samples were pressed to each other, and the powder was milled in smooth movements until a uniform coating was formed.

**Figure 1:**
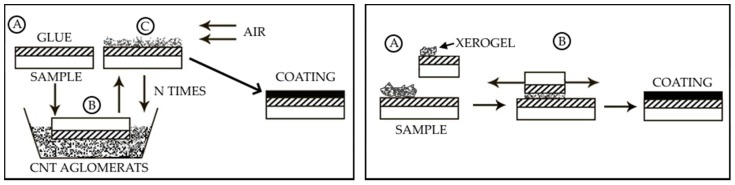
Two application methods: on the left is the method of dipping the sample into agglomerates (Stage A—applying the adhesive layer to the surface, B—dipping the sample into agglomerates, C—blowing off non-fixed CNTs), on the right is the xerogel grinding method (Stage A—applying xerogel powder to the prepared sample, stage B—grinding the powder until a homogeneous layer is formed).

Moreover, a series of samples was obtained through sticking CNT agglomerate to the carbon adhesive tape without grinding in order to identify the main factors determining the properties of the coating. The agglomerate powder was sprinkled over the viscous coating over and over; agglomerates that remained unstuck deflated. The process was repeated until a uniform layer was formed. Another series of samples made of CNT agglomerates was obtained similarly to the xerogel coating. For visual reference, Figure 1 shows both methods of applying.

The coated sample was a 3 cm by 3 cm. Similar samples with a steel base were used to measure superhydrophobic and anti-icing properties. A polyacrylic insulator was used as a basis of the sample to determine the resistivity of the coating.

### 2.3. Wetting Angle Measurements

The hydrophobicity of the final product surface was measured on a Drop Shape Analyzer—DSA20E (KRÜSS GmbH, Hamburg, Germany). The measuring method of the water contact angle and slip start angle is sessile drop method. For that purpose, we measured the characteristics in 10 random spots of 3 similar samples, and after that the obtained data were averaged. Distilled water served as a liquid. The volume of the droplet was 10 microliters.

### 2.4. Icing

**Figure 2:**
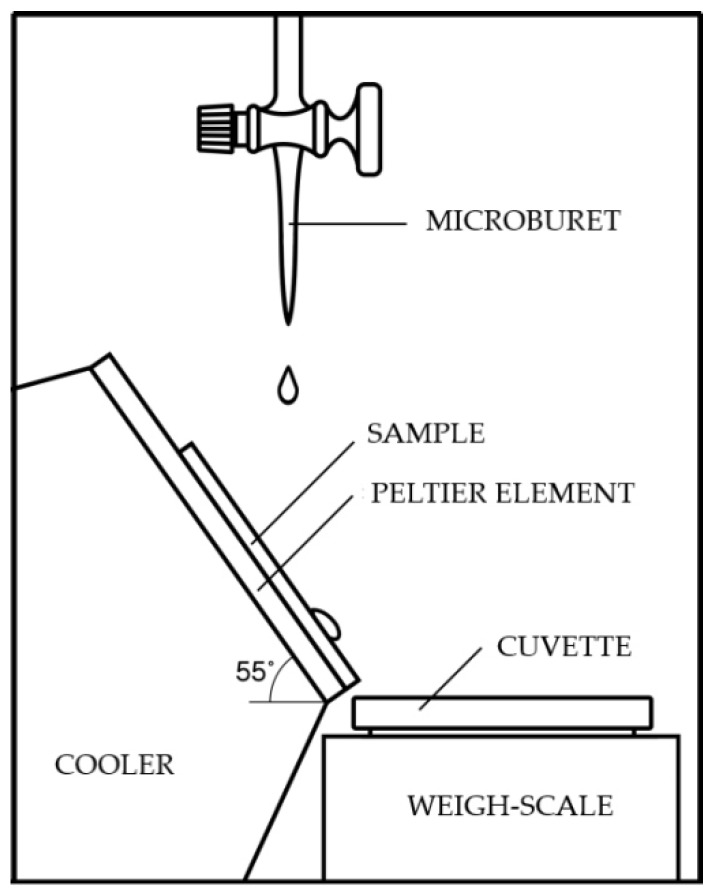
Schematic illustration of the facility for measuring icing speed.

Figure 2 demonstrates schematic illustration of the facility for measuring icing speed. Two samples (regular stainless steel and stainless steel with a superhydrophobic coating) were fixed on a Peltier element and inclined to the horizon at an angle of 55°. The samples were cooled down to the temperatures of −5 °C and −15 °C. A series of droplets with a total volume of 0.1 ± 0.05 mL of water was put in the center of a sample using a microburet. The droplets rolled off the surface of a sample into a special cuvette, their mass was measured to within 0.002 g. Then the next portion of water was put on the sample. The cycle was repeated multiple times, the mass of the water in the cuvette determined the mass of the liquid frozen on the surface of the sample. The experiment was repeated twice, and then the dependence of the icing mass on the mass of poured droplets was averaged. The coating for the study of anti-icing properties was obtained from xerogel based on “Taunit-MD” CNTs and ethanol.

### 2.5. SLIPS Effect

The samples were moistened by lubricant (perfluorodecalin), and then the water contact angle and the slip start angle on the processed surface were measured. The amount of lubricant was x g per cm^2^. This procedure was applied both to qualitative samples based on Taunit-MD and ethanol and to several samples with defective coatings. “Defective” samples signify samples with various mechanical surface damage that caused a sharp increase of the slip start angle. As a result, the samples lost superhydrophobicity. The damage was inflicted because of a violation sample production method or appeared during its handlingThe slip start angle on defective samples was measured in 10 different spots of each sample and after that the data were averaged.

### 2.6. Conductivity of the Samples

The surface resistivity of the samples was measured through direct evaluation using an OA 3201 laboratory ohmmeter (ElectronPribor ltd, Fryazino, Russia). Electrodes were pressed against the opposite sides of the coating. The measurements were carried out 10 times for various samples of the same type and after that the data were averaged. The coating was obtained from xerogel based on “Taunit-MD” CNTs and ethanol.

### 2.7. Scanning Electron Microscopy

A Tescan VEGA 3 SBH scanning electron microscope (TESCAN orsay holding, a.s., Brno, Czech Republic) was used for obtaining pictures. The microscope is equipped with a tungsten thermoemission cathode. The pictures were obtained with a voltage of 5 kV and an Everhart-Thornley detector of secondary electrons (YAG crystal) in a high-vacuum mode (9 × 10^−3^ Pa).

## 3. Results and Discussion

### 3.1. Hydrophobic Coatings Based on Xerogel and Agglomerates

**Figure 3:**
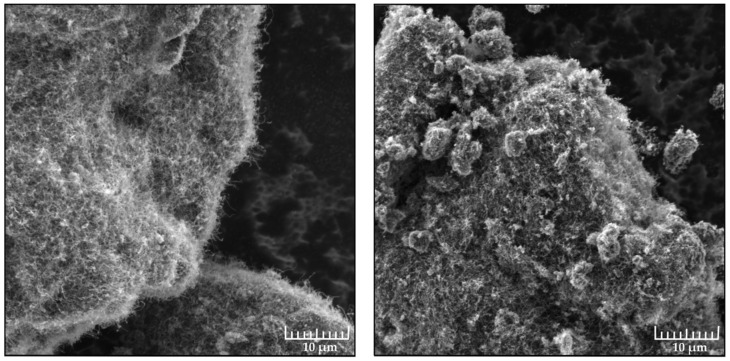
Hydrophobic coating based on Taunit CNT agglomerates. Left side shows application through sticking, right side demonstrates application through grinding. The image was obtained through a Tescan Vega 3 scanning electron microscope.

After production carbon nanotubes assume the form of black powder consisting of agglomerates—lumps of closely intertwined carbon nanotubes. A protective coating based on agglomerates (the data are provided in Table 2) has rather variegated properties. This is demonstrated by a large value spread between the water contact angle and the slip start angle. What is also worth mentioning is the fact that properties of the surface depend heavily on the method of application. Figure 3 shows that application through grinding leads to modification of the agglomerate surface which, in turn, leads to amplification of the lotus effect. The measurements show that the structure of the surface of CNT agglomerates has a greater effect than their geometrical characteristics.

It is worth noting that in various factory batches the structure and the density of agglomerates—and, therefore, the properties of the coating—can vary significantly. CNT agglomerates have a low density and are prone to external influence. In order to nullify these drawbacks a decision was made to study the coating made of milled xerogel. The morphology and the properties of the xerogel are determined by the conditions of its drying and, therefore, easy to control. The structure of the xerogel is far less variegated, which leads to improvement of its mechanical properties and an increase in uniformity of the properties of the surface.

**Figure 4:**
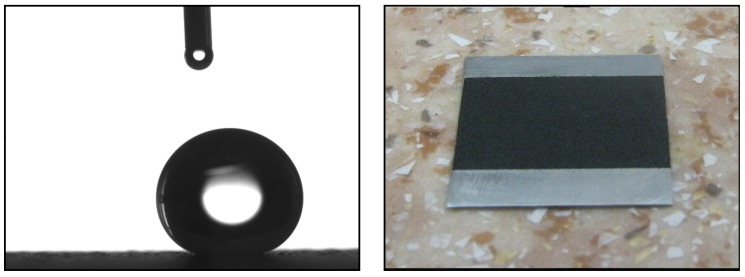
On the left is the image of drop on the best sample (water contact angle 159 degrees), on the right is the xerogel applied on the metal plate.

**Figure 5:**
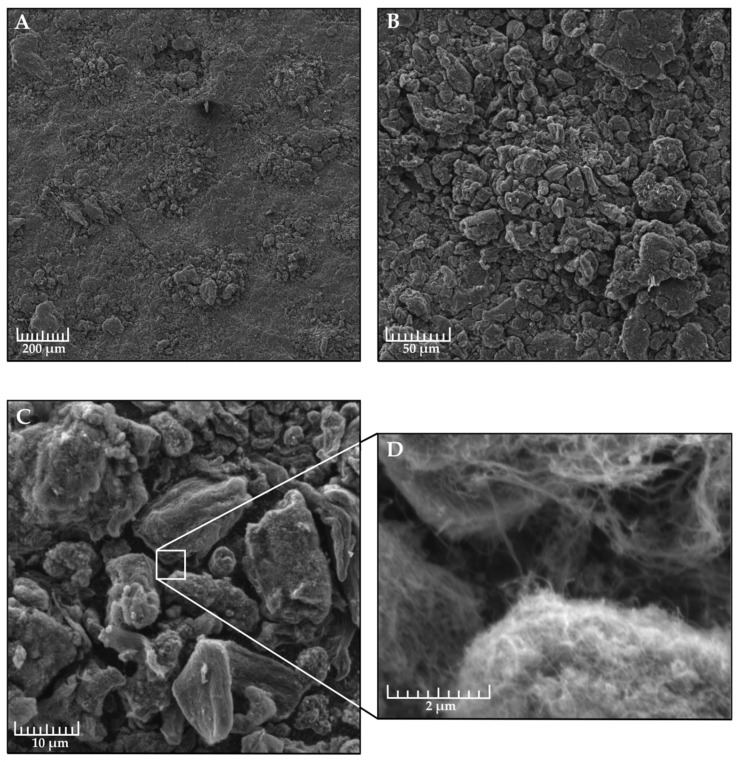
Superhydrophobic surface at various zoom degrees. The sample is made of xerogel based on Taunit-MD CNTs and ethanol, the fraction size equals 0.125–0.100 mm. Section **A**—200 times magnification, a coating defect is visible at the top. At these points, the development of icing begins. Section **B**—magnification 1000 times. Section **C**—10,000 times. Section **D**—an increase of 40,000 times, individual CNTs are visible. The image was obtained through a Tescan Vega 3 scanning electron microscope.

Table 3 shows the results of a measurement of hydrophobic properties of a coating made of milled xerogel depending on the fractional composition and the polarity of the dissolvent. Figure 4 shows the image of drop on the best sample with the highest water contact angle, obtained using the drop shape analyzer described above and the xerogel applied on the metal plate. Figure 5 demonstrates the SEM image of the optimal fraction in regards to the water contact angle.

An increase in the water contact angle and a decrease in the slip start angle as the fraction size is reduced to 0.100–0.125 mm is typical for all samples. In addition, uniformity of the properties of the surface increases. However, upon further reduction of the fraction size the properties of the surface begin to deteriorate—we explain it the following way: in order to shred the xerogel further a longer milling is required, during which the morphology of xerogel particle surface deteriorates—the lotus effect becomes less noticeable.

Using a more polar dissolvent—ethanol—enabled the improvement of the surface characteristics. A similar effect was observed in Liu’s et al. work [17]. It was discovered that the use of a “bad”, more polar dissolvent leads to the appearance of a more developed surface. In such an environment long, non-polar molecules are more prone to agglomeration and “huddle” into nanoglobules. Moreover, a certain deterioration was observed in the mechanical durability of the xerogel particles. Naturally, the CNTs rolled into nanoglobules have more contact points between each other than with the surrounding CNTs. This leads to an increase in brittleness of the particles. Reduction of the CNT aspect ratio has a similar effect on the xerogel durability. The xerogel based on Taunit CNTs turned out too brittle to be used, which is why its data are not provided.

### 3.2. Icing

The experiment shows that applying the coating allows to significantly reduce the frosting of the sample. Upon decreasing the temperature, the rate of ice-forming on the surface increases, but still remains several times lower than that of a sample without a coating. Due to a water contact angle and the homogeneity of the surface the droplet rolls off the coating before it can cool down and freeze.

**Figure 6:**
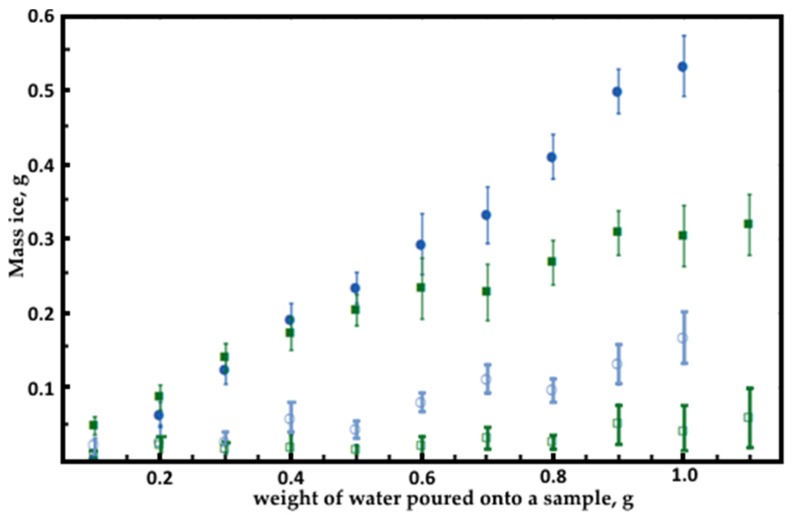
Icing mass on samples depending on the amount of water poured onto them. Filled marks signify steel samples; empty marks denote samples with coatings of xerogel based on Taunit-MD and ethanol (with a fraction of 0.125–0.100 mm). Blue marks correspond to samples cooled down to −15 °C, green marks represent samples cooled down to −5 °C. The data presented are an average of three measurements.

The process of surface frosting is nondeterministic. A falling droplet can bring formed ice with it or freeze completely on a surface defect. This leads to certain curvatures in Figure 6.

### 3.3. SLIPS Effect

After processing xerogel with a lubricant properties of the surface are determined by the interaction of the lubricant and the droplet. The water contact angle declines abruptly, but the slip start angle of the droplet becomes significantly smaller, the droplet barely lingers on the surface defects. As a matter of fact, the surface remains self-cleaning. Table 4 shows the data of slip start angle change for 7 defective samples based on Taunit-MD and ethanol. Perfluorodecalin was used as a lubricant. Perfluorodecalin posesses all properties of perftorans—minimum affinity for polar and non-polar liquids, biological passiveness and ecological safety. Perftorans are considered the optimal lubricant for slip surfaces.

The worse the slip angle initially was, the more significant is its improvement while using a lubricant. Covering the spots damaged during the operation with a lubricant can serve as a prompt repair. The same pattern can allow using larger xerogel fractions for creating coatings.

A lubricant can also be picked in a certain way in order to improve the anti-icing properties of the surface or perform the biocidal function. Table 5 shows the data on changing the properties of a good sample during the application and drying of the lubricant. It is clear that deterioration of the properties of the surface during the lubricant evaporation is insignificant.

In order to increase the retention time of the lubricant, CNTs can be functionalized by choosing the type of functional groups that has the highest affinity for the lubricant. An effective functionalization procedure for perfluoranes is provided in [13]. Moreover, it is desirable to use more viscous liquids with high molecular masses.

### 3.4. Conductivity of the Coating

Results of the measurements are presented in Table 6. A decrease in the size of the xerogel fraction leads to an increase in coating resistance. Coating resistance allows static removal or heating of the coating by the Joule-Lenz effect.

## 4. Conclusions

In this study, a simple approach was proposed to obtain a superhydrophobic surface by applying CNT agglomerates from milled xerogel. Hierarchical multiscale was formed by means of both CNT agglomerates at the microscale and MWCNTs themselves at the nanoscale. This allowed us to obtain a superhydrophobic coating with a water contact angle of approximately 156° at the droplet inclination angle of 3.6°. During tests the coating held a droplet for nearly 4 h before its drying.

According to its characteristics, the proposed approach to create a superhydrophobic coating based on carbon nanoparticles is competitive with most of the methods presented in the review. Moreover, the suggested method of application (sticking prepared xerogel particles with a specific morphology to the prepared surface) is very different from others and gives a complex effect.

Growing a nanotube forest is a complex task requiring specialist equipment, high temperatures, and it can be done with a limited amount of materials. Transferring the completed “CNT forest” to the protected surface is also a difficult task. Moreover, vertically-oriented CNTs rarely touch each other and, as a result, the conductivity and sorption properties of the coating are not as high as desired. In the case of condensation of moisture or frost on the side surface of the CNT, the array also loses superhydrophobic or anti-icing properties.

A different approach associated with the spraying of CNT dispersions in a polymer is certainly convenient to apply, but their high conductivity is guaranteed only at high concentrations of CNTs. In [25], this problem was solved by the addition of soot. The sorption capacity of such coatings is low as well.

Our approach allows you to work with any type of surface (you only need to choose an adhesive layer with an acceptable level of adhesion) and does not require special conditions for the growth of CNTs. The complexity of the approach does not significantly exceed the methods based on the application of CNT dispersions, but it allows to obtain a higher level of conductivity and sorption capacity.

An approach closest to ours was used in [29], where silica aerogel armed with CNTs was produced. CNTs ensure reinforcement of the aerogel, and the aerogel itself has a high sorption capacity. However, xerogel made only from carbon nanoparticles promises higher conductivity.

Our work is also related to the idea suggested in [19]. In our work, particles of milled xerogel covered with nanotubes projecting in all directions act as a microrelief on a silicon wafer.

Fluorination or coating with fluoropolymers in a way similar to [3] may lead to an improvement in the properties of the coating, but will increase its production technology by an additional stage, which will increase the complexity and cost of the coating.

Other application methods, such as those described in [25] where a CNT conductive coating was also obtained, cannot create a combination of micro- and nanoscale roughness necessary to obtain a superhydrophobic coating. Thus, we can say that the proposed approach allows you to effectively combine all three methods of creating hydrophobicity and conductivity sufficient for heating with reasonable application difficulties. The grinding method is the preferable method of application. It ensures a higher effectiveness of the coating and homogeneity of its properties.

An analysis of the experimental data allows to make the following conclusions:

1. One of the main factors that determine the properties of a coating is the relief of the particle surface made of carbon nanotubes. The morphology of the surface of CNT agglomerates depends heavily on the conditions of synthesis of the CNTs and the conditions of their drying, and can vary in different batches. Furthermore, the surface structure can change under mechanical effect and chemical processing;
2. The use of milled xerogel powder allows to obtain a hydrophobic surface with a high homogeneity of properties that is more resistant to mechanical effect. The morphology of xerogel is determined mainly by drying conditions and milling duration;
3. During the production of xerogel the use of more polar dissolvents allows to obtain particles with a more developed nanorelief of the surface, but with worse mechanical properties;
4. It is preferable to use CNTs with a high aspect ratio—they do not only produce better results, but also allow to improve the mechanical properties of xerogel due to a larger number of contact points between the CNTs. It is also possible to improve the mechanical properties of a coating by introducing a binding hydrophobic polymer into the xerogel. The production method of xerogels is widely known; examples can be seen in [26,27,28,29];
5. CNTs possess a high sorption capability, which allows to use the suggested coating to reproduce the SLIPS effect. Meanwhile the lubricant can have anti-icing or biocidal properties. The lubricant can also be used for prompt repair—when applied to damaged spots, it reduces the slip start angle of the droplet;
6. The high hydrophobicity of the coating allows to decelerate frosting formation. The droplet slips from the coating before it can freeze. As a result, the coating works best at temperatures close to zero, upon further cooling the pace of frosting formation increases.

Good conductivity will allow to remove a static charge and to carry out induction heating of the surface. This effect can be applied, for example, to wind generators blades working under icing conditions. The resulting material based on CNT can provide superhydrophobic properties. No special conditions are required for its application to protected objects, and the possibility of applying the coating to a certain material itself is determined by the existence of a viscous glue for this material with good adhesion to carbon.

## Figures and Tables

**Table 1:** Characteristics of MWCNTs of various grades from the manufacturer.

Characteristic	Taunit	Taunit–M	Taunit–MD
Outer diameter, nm	20–50	10–30	8–30
Internal diameter, nm	10–20	5–15	5–15
Length, μm	≥2	≥2	≥20
The total amount of impurities, %	≤10	≤5	≤5
Specific surface, m^2^/g	≥160	≥270	≥270
Bulk density, g/cm^3^	0.3–0.6	0.025–0.06	0.025–0.06
Cost, dollar per gram	0.2	1.5	1.5

**Table 2:** Water contact angle of a droplet on a protective coating out of CNT agglomerates.

Model	Application through Sticking	Application through Grinding
Taunit	110.7° ± 10.8°	135.1° ± 4.8°
Taunit-М	121.1° ± 8.3°	143.7° ± 4.1°
Taunit-МD	127.0° ± 6.4°	146.0° ± 3.4°

**Table 3:** Influence of the fraction size and the dissolvent type on the hydrophobic properties of the coating.

Xerogel Material	Taunit-M and Isopropanol		Taunit-MD and Isopropanol		Taunit-M and Ethanol	
Fraction Size, mm	Water Contact Angle, °	Slip Start Angle, °	Water Contact Angle, °	Slip Start Angle, °	Water Contact Angle, °	Slip Start Angle, °
0.350–0.250	143.5 ± 3.3	15.0 ± 8.5	144.8 ± 2.1	10.5 ± 5.0	151.5 ± 2.2	12.6 ± 5.6
0.250–0.140	145.9 ± 1.9	12.7 ± 6.2	147.2 ± 1.9	9.1 ± 5.2	153.8 ± 2.8	5.1 ± 1.5
0.140–0.125	146.3 ± 1.2	9.3 ± 4.4	148.3 ± 1.7	8.0 ± 1.0	155.9 ± 1.5	3.7 ± 1.8
0.125–0.100	147.2 ± 0.5	5.1 ± 2.2	150.5 ± 0.6	4.4 ± 0.4	155.6 ± 1.1	3.6 ± 1.2
0.100–0.071	146.3 ± 1.1	5.3 ± 2.8	148.8 ± 0.9	4.7 ± 1.5	155.6 ± 1.0	3.7 ± 1.1

**Table 4:** Slip start angle of mechanically damaged samples before and after applying the perfluorodecalin as a lubricant.

**Slip Start angle, °**	Before Applying the Lubricant	The droplet doesn’t slide	88.2 ± 12.5	41.9 ± 15.7	36.2 ± 13.2	25.2 ± 15.1	19.9 ± 5.7	14.3 ± 4.5
	After Applying the Lubricant	6.4 ± 2.5	6.3 ± 1.2	4.5 ± 1.4	2.5 ± 1.1	6.3 ± 2.1	7.0 ± 1.8	3.4 ± 1.7

**Table 5:** Properties of a sample with a hydrophobic coating of Taunit-MD and ethanol (a fraction of 0.100–0.125 mm) before and after applying the lubricant and after drying the lubricant.

Sample	Water Contact Angle	Slip Start Angle
Before applying perfluorodecalin	157.7 ± 1.4	4.7 ± 1.8
With perfluorodecalin	102.0 ± 0.9	2.5 ± 0.6
After drying perfluorodecalin	154.5 ± 2.7	5.6 ± 1.9

**Table 6:** Dependence of the resistance of the sample on the size of the xerogel fraction.

**Fraction size, mm**	0.100–0.071	0.125–0.100	0.140–0.125	0.250–0.140	0.350–0.250
**Resistance, kOhm/sm^2^**	0.47 ± 0.03	0.44 ± 0.05	0.35 ± 0.03	0.34 ± 0.01	0.29 ± 0.02
